# Potential relevance between soybean nitrogen uptake and rhizosphere prokaryotic communities under waterlogging stress

**DOI:** 10.1038/s43705-023-00282-0

**Published:** 2023-07-11

**Authors:** Tengxiang Lian, Lang Cheng, Qi Liu, Taobing Yu, Zhandong Cai, Hai Nian, Martin Hartmann

**Affiliations:** 1 The State Key Laboratory for Conservation and Utilization of Subtropical Agro-Bioresources, South China Agricultural University, Guangzhou, Guangdong China; 2 The Key Laboratory of Plant Molecular Breeding of Guangdong Province, College of Agriculture, South China Agricultural University, Guangzhou, Guangdong China; 3 Institute of Agricultural Sciences, ETH Zurich, Zurich, Switzerland

**Keywords:** Microbial ecology, DNA sequencing, Stable isotope analysis

## Abstract

Waterlogging in soil can limit the availability of nitrogen to plants by promoting denitrification and reducing nitrogen fixation and nitrification. The root-associated microorganisms that determine nitrogen availability at the root-soil interface can be influenced by plant genotype and soil type, which potentially alters the nitrogen uptake capacity of plants in waterlogged soils. In a greenhouse experiment, two soybean genotypes with contrasting capacities to resist waterlogging stress were grown in Udic Argosol and Haplic Alisol soils with and without waterlogging, respectively. Using isotope labeling, high-throughput amplicon sequencing and qPCR, we show that waterlogging negatively affects soybean yield and nitrogen absorption from fertilizer, atmosphere, and soil. These effects were soil-dependent and more pronounced in the waterlogging-sensitive than tolerant genotype. The tolerant genotype harbored more ammonia oxidizers and less nitrous oxide reducers. Anaerobic, nitrogen-fixing, denitrifying and iron-reducing bacteria such as *Geobacter/Geomonas*, *Sphingomonas*, *Candidatus Koribacter*, and *Desulfosporosinus* were proportionally enriched in association with the tolerant genotype under waterlogging. These changes in the rhizosphere microbiome might ultimately help the plant to improve nitrogen uptake under waterlogged, anoxic conditions. This research contributes to a better understanding of the adaptability of soybean genotypes under waterlogging stress and might help to formulate fertilization strategies that improve nitrogen use efficiency of soybean.

**Figure:**
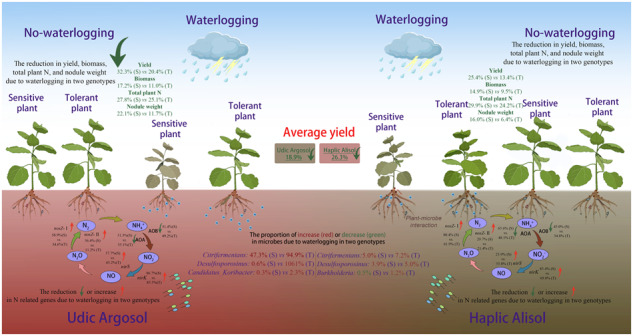
Schematic representation of the effects of waterlogging on nitrogen uptake and rhizosphere microbiota in dependence of soil type and soybean genotype.

Schematic representation of the effects of waterlogging on nitrogen uptake and rhizosphere microbiota in dependence of soil type and soybean genotype.

## Introduction

In recent years, the frequency of extreme weather events, including heavy rainfall and the associated soil waterlogging events, which could last from a few hours to several days, has increased [1, 2]. In legumes, nitrogen (N) uptake and crop yield are largely determined by symbiotic N_2_ fixation and N availability in soil, which are negatively affected by waterlogging stress [3–5]. Previous research has shown that plants with a high N uptake efficiency can mitigate the negative impact of waterlogging [5]. Certain plant genotypes have developed various mechanisms to increase N uptake during waterlogging, including the production of antioxidant enzymes and sugars, and the formation of more adventitious roots and aerenchyma in the root system [6–8].

Root-associated microorganisms play a key role in regulating plant N uptake in waterlogged soils [9, 10]. Waterlogging increases the water-filled pore space, which reduces oxygen availability and decreases heterotrophic respiration, symbiotic N_2_ fixation, and nitrification [11–13]. The hypoxic conditions in waterlogged soils reduce the activity of ammonia-oxidizing bacteria (AOB) and archaea (AOA) leading to a reduction in nitrification rates [13]. In contrast, waterlogging also promotes soil N losses from the system through runoff, leaching and anaerobic processes like denitrification, ultimately resulting in reduced crop productivity [2, 13]. It is not yet well understood if different genotypes that are sensitive or tolerant to waterlogging stress shape these N-cycling guilds and the associated processes in the rhizosphere differently.

Soil physico-chemical properties including texture, porosity, and pH are important determinants of how soils respond to waterlogging [14, 15]. Soil texture determines the water holding capacity with clayey soils becoming waterlogged more easily due to their higher water-holding capacity and slower permeability [14]. Soil porosity determines the water movement and aeration whereas soil with higher porosity have better drainage and higher aeration [16]. Finally, pH influences nutrient availability in the soil [17] which might modulate the response of plants to waterlogging stress. Differences in these properties might therefore largely shape the effects of waterlogging on plants and associated microbiomes.

Moreover, tolerant plant genotypes can recruit specific microorganisms to resist biotic and abiotic stresses [18, 19]. For example, it has been shown that microorganisms like *Flavobacterium* isolated from the rhizosphere of disease-resistant tomato plants can suppress disease symptoms of susceptible genotypes [18]. Our previous research has indicated that aluminum-tolerant soybeans might recruit some microbial taxa such as *Tumebacillus*, *Burkholderia, Penicillium*, and *Cladosporium* that could help mitigating aluminum toxicity [20, 21]. Research on waterlogging tolerance has so far largely focused on understanding and improving the plant genetic components, while the role of rhizosphere microorganisms, especially those involved in N cycling, has rarely been studied [22]. Exploring the potential role of rhizosphere microorganisms in enhancing plant tolerance to waterlogging could open new avenues for stress management of crops.

This study aimed to evaluate the effect of soybean genotypes with different sensitivities to a 3-day waterlogging stress on N acquisition and the rhizosphere microbiome in Udic Argosol and Haplic Alisol soils. The amount of plant N derived from atmospheric N_2_, N fertilizer and soil mineralization was assessed using the ^15^N dilution method [23, 24]. The abundance of nitrifiers and denitrifiers was estimated using quantitative PCR (qPCR) of the *amoA*, *nirS*, *nirK*, and *nosZ* genes, and the rhizosphere prokaryotic community structure was determined by 16S rRNA gene sequencing. Because the Udic Argosol had lower porosity and higher clay content than the Haplic Alisol, we hypothesized that the waterlogging effects will be more pronounced in the Udic Argosol, which will result in a stronger reduction in N uptake by soybean plants when compared to the Haplic Alisol. We further hypothesized that the waterlogging-tolerant soybean genotype can enrich specific N-cycling microbes under waterlogging stress that might help with increasing N availability and acquisition.

## Methods and material

### Soil type and plant materials

Soils were collected down to 15 cm from two soybean cultivation areas of Suixi County (110°25′N, 21°32′E) and Yingde County (113°40′N, 24°18′E), Guangdong province of China, which were classified as Udic Argosol and Haplic Alisol, respectively, according to USDA soil taxonomy. The soil chemical characteristics were: pH 5.3, 40.2% porosity, and 40.2% clay content for the Udic Argosol (U) and pH 7.1, 55.4% porosity, and 13.6% clay content for the Haplic Alisol (H), respectively. Moreover, two different soybean (*Glycine max L*.) genotypes that were shown to be either tolerant (Qihuang34) or sensitive (Jidou17) to waterlogging stress were investigated in this study [25]. Qihuang34 has been shown to activate enzymatic pathways related to glycolysis and gluconeogenesis to produce adenosine triphosphate (ATP) for plant survival under anaerobic conditions and to downregulate lignin biosynthesis pathways leading to plant softening under waterlogging [25]. Additionally, compared with sensitive lines, waterlogging-tolerant soybean genotypes have more aerenchyma and adventitious roots [26], and less sucrose [8] and endogenous abscisic acid (ABA), which could enhance waterlogging tolerance through the control of energy conservation via the glycolytic system [27].

### Greenhouse experiments and ^15^N Labeling

A pot experiment with a randomized block design featuring nine replications for each treatment (i.e., soil type, soybean genotype, waterlogging stress), was installed in a greenhouse of the South China Agricultural University in Guangzhou, China. Each pot was filled with 8 kg of soil and seeded with eight soybean seeds of uniform size. Ten days after sowing, some seedlings were removed to keep the three best growing soybean plants per pot. Ca(NO_3_)_2_ labeled with ^15^N at 5 atom percent was added to the soil as the N fertilizer at a rate of 100 mg N kg^−1^. To calculate the amount of biological nitrogen fixation, a soybean mutant incapable of inducing nodulation was planted under the different waterlogging conditions and soil types as reference species without the ability of fixing atmospheric N_2_ [23, 28]. The growth conditions were set to a photoperiod of 14/10 h light/dark cycle and an average temperature of 28 °C and 20 °C during day and night, respectively. When soybeans reached the flowering stage, water was added to the pots up to 4 cm above the soil level for 3 days to induce waterlogging stress, whereas the control plants were maintained at 80% field capacity, which corresponds to 20% water content. A 3-day period was chosen to assess short-term effects of waterlogging. The experimental design is shown in Supplementary Fig. S1.

Rhizosphere soil samples were collected immediately following a 3-day waterlogging period, with the three plants per pot combined into one sample, by gentle shaking the harvested root system and transferring the fine roots (~20 g) with the remaining adhering soil to a 50 ml centrifuge tube filled with phosphate-buffered saline (PBS). After 2 min shaking and then 10 min centrifugation at 13,000 rpm, five grams of the rhizosphere soil pellet were stored at −80 °C for DNA extraction and the remaining pellet was kept at 4 °C for soil physical and chemical analyses. Then, the soybean shoot and root were collected for the biomass measurement. Soybean plants of three out of the nine pots per treatment were kept and harvested at the R8 stages (maturity, 120 days after sowing) to measure the yield.

In this study, the natural ^15^N abundance in the atmosphere (0.3663 atom% ^15^N) was referenced to calculate the atom% ^15^N [29]. The content of plant N derived from fertilizer (N_f_), N_2_ derived from the atmosphere (N_a_) and soil (N_s_) were calculated as follows [28, 30]:

$${{{{{{{{{\rm{N}}}}}}}}}}_{{{{{{{\mathrm{f}}}}}}}} = {{{{{{{{{\rm{N}}}}}}}}}}_{{{{{{{{\mathrm{plant}}}}}}}}}\left( {{{{{{{{\mathrm{mg}}}}}}}}\,{{{{{{{\mathrm{plant}}}}}}}}^{ - 1}} \right) \times {{{{{{{{{\rm{N}}}}}}}}}}\,{{{{{{{\mathrm{atom}}}}}}}}\% ^{15}{{{{{{{\mathrm{N}}}}}}}}\,{{{{{{{\mathrm{excess}}}}}}}}\,{{{{{{{\mathrm{in}}}}}}}}\,{{{{{{{\mathrm{plant}}}}}}}}/{{{{{{\rm{N}}}}}}}\,{{{{{{{\mathrm{atom}}}}}}}}\%$$

$${{{{{{{{{\rm{N}}}}}}}}}}_{{{{{{{\mathrm{a}}}}}}}}\left( {{{{{{{{\mathrm{mg}}}}}}}}\,{{{{{{{\mathrm{plant}}}}}}}}^{ - 1}} \right) \,=\,	 \left\{ {1{{{{{{{\mathrm{ }}}}}}}} - {{{{{{{\mathrm{ }}}}}}}}\left[ {{{{{{{{\mathrm{atom}}}}}}}}\% ^{15}{{{{{{{\mathrm{N}}}}}}}}\,{{{{{{{\mathrm{excess}}}}}}}}\,\,\left( {fs} \right)/{{{{{{{\mathrm{atom}}}}}}}}\% ^{15}{{{{{{{\mathrm{N}}}}}}}}\,{{{{{{{\mathrm{excess}}}}}}}}\left( {nfs} \right)} \right]} \right\}\\ 	\times {{{{{{{{{\rm{N}}}}}}}}}}_{{{{{{{{\mathrm{plant}}}}}}}}}\left( {{{{{{{{\mathrm{mg}}}}}}}}\,{{{{{{{\mathrm{plant}}}}}}}}^{ - 1}} \right)$$

where *fs* is N_2_ fixing system, *nfs* is non-fixing (soybean mutants) system, and N_plant_ is the N content of each plant.

$${{{{{{{{{\rm{N}}}}}}}}}}_{{{{{{{\mathrm{s}}}}}}}} = {{{{{{{{{\rm{N}}}}}}}}}}_{{{{{{{{\mathrm{plant}}}}}}}}}\left( {{{{{{{{\mathrm{mg}}}}}}}}\,{{{{{{{\mathrm{plant}}}}}}}}^{ - 1}} \right) - {{{{{{{{{\rm{N}}}}}}}}}}_{{{{{{{\mathrm{f}}}}}}}} - {{{{{{{{{\rm{N}}}}}}}}}}_{{{{{{{\mathrm{a}}}}}}}}$$

### Plant and soil chemical analysis

The plant root N contents were measured using an Vario EL III Elemental Analyzer (Elementar Scientific Instruments, Hanau, Germany). Soil pH was measured in aqueous solution using a FE20-FiveEasy™ pH meter (Mettler Toledo, Giessen, Germany). The ^15^N/^14^N ratio was measured using an Deltaplus isotope ratio mass spectrometer (Finnigan MAT GmbH, Bremen, Germany). Soil organic carbon (SOC) was measured by combustion using an SSM-5000A analyzer (Shimadzu, Kyoto, Japan). Available K (AK) was quantified using an ICPS-7500 inductively coupled plasma-atomic emission spectrometry (Shimadzu, Japan). Total soil phosphorus (TP), available phosphorus (Olsen-P), nitrate (NO_3_^−^) and ammonium (NH_4_^+^) was measured using a San^++^ continuous flow analytical system (Skalar, Breda, The Netherlands).

### DNA extraction and 16S rRNA gene sequencing

Total nucleic acids were extracted from 0.5 g soil using the Fast DNA SPIN Kit for Soil (MP Biomedicals, Santa Ana, CA, USA) according to the manufacturer’s instructions and quantified on a Nanodrop 2000 spectrophotometer (Thermo Fisher Scientific, Waltham, MA, USA). Primers of 515F and 909R with variable 12 bp barcode sequences were used to amplify the V4 region of the 16S rRNA gene [31]. PCR amplification was carried out in a 20 µl reaction volume including 15 µl PCR SuperMix (Takara, Dalian, China), 20 µM forward and reverse primers, and 10 ng of template DNA. Thermocycling conditions consisted of an initial denaturation step at 95 °C for 60 s followed by 30 cycles of denaturation at 94 °C for 1 min, annealing at 55 °C for 1 min, and elongation at 75 °C for 2 min, with a final elongation cycle at 75 °C for 5 min. V4 amplicons were sequenced using the Illumina MiSeq PE250 platform at Majorbio Bio-pharm Technology Co., Ltd (Shanghai, China). Raw sequence data are available at the NCBI sequence read archive (SRA) under the accession number PRJNA723464.

### Quantitative PCR of bacterial and archaeal marker genes

Gene copies of the bacterial and archaeal 16S rRNA genes, bacterial and archaeal *amoA* genes, *nirS*, *nirK* and *nosZ* clade I and II genes were determined by SYBR Green based qPCR assays on an ABI 7900 system (Thermo Fisher Scientific). Details of primer sequences and thermocycling conditions are described in Supplementary Table S1. A plasmid of known concentration was spiked into the soil DNA extracts and qPCR amplified using vector-specific primers SP6 and T7 to assess potential variability in amplification inhibition across the extracts. Bacterial and archaeal 16S rRNA genes were amplified with primers 515F/909R [31] and 967F/1060R [32], respectively. Bacterial and archaeal *amoA* genes were amplified with primers of amoA-1F/amoA-2R and CrenamoA23f/CrenamoA616r, respectively [33]. Genes *nirS* and *nirK* were amplified with primers of nirS-efF/nirS-efR [34] and nirKC2F/nirKC2R [35], respectively, whereas the *nosZ-*I and *nosZ-*II genes were amplified with primers of NosZ2f/NosZ2r [36], and nosZIIF/nosZIIR [37], respectively. Standard curves were constructed using plasmid DNA containing [33] the target gene diluted from 10^9^ to 10^2^ with 10-fold serial dilutions. Amplification efficiencies were 98.6 (*R*^2^ = 0.997) for the bacterial 16S rRNA gene, 97.4 (*R*^2^ = 0.995) for the archaeal 16S rRNA gene, 97.2 (*R*^2^ = 0.995) for the bacterial *amoA* gene, 98.7 (*R*^2^ = 0.997) for the archaeal *amoA* gene, 96.4 (*R*^2^ = 0.994) for the *nirS* gene, 95.8 (*R*^2^ = 0.992) for the *nirK* gene, 95.5 (*R*^2^ = 0.998) for the *nosZ* clade I gene, and 96.5 (*R*^2^ = 0.994) for the *nosZ* clade II gene. PCR amplification of these genes was carried out in a 20 µl reaction volume including 15 µl SYBR Green Master Mix (Takara, Dalian, China), 20 µM forward and reverse primers, and 10 ng of template DNA. Melting curve analysis was performed to check the specificity of the primers.

### Bioinformatics and statistics

Sequence data were processed using a customized pipeline based on VSEARCH v.2.21.1 [38] as described previously [39]. In brief, PhiX contaminants were removed by aligning the reads against the PhiX genome (NC_001422.1) using Bowtie2 v.2.4.2 [40]. PCR primers were trimmed using Cutadapt v.3.4 allowing one mismatch [41]. Paired-end reads were merged and quality-filtered allowing a maximum expected error of one using the functions *fastq_mergepairs* and *fastq_filter* implemented in VSEARCH, respectively [42]. Reads were delineated into amplicon sequence variants (ASVs) using the functions *derep_fulllength* and *unoise3* implemented in VSEARCH [43]. Potentially chimeric reads were identified and removed using the function *uchime3_denovo* implemented in VSEARCH [44]. The sequences were then tested for ribosomal features using Metaxa2 v.2.2.3 [45]. The quality filtered reads were mapped against the verified ASV sequence to obtain the final ASV table using the *usearch_global* function implemented in VSEARCH with an identity threshold of 97%. Taxonomic classification of the ASV sequences was performed using the Sintax algorithm [46] implemented in VSEARCH against the SILVA v.138 database using a bootstrap cut-off value of 0.8. ASVs not assigned at the domain level or assigned to organelle structures (chloroplasts and mitochondria) were removed from the final ASV table.

Statistical analyses were conducted in R v.4.2.1 [47]. The effects of soil type, waterlogging stress and soybean genotype on soil properties, plant properties, and univariate prokaryotic properties (i.e., gene copy numbers and alpha diversity metrics) were assessed by factorial ANOVA followed by Tukey’s HSD. Normality of residuals and homoscedasticity were confirmed with the Shapiro–Wilk and Levene tests implemented in the *stats v.4.2.1* and *car v3.1.0* packages in R. Alpha-diversity (observed richness, Pielou’s evenness and Shannon diversity) and beta-diversity (Bray–Curtis’s dissimilarity) properties were calculated using the *diversity* and *vegdist* functions of the R package vegan v.2.6.2 [48] based on an iterative (100 iterations) subsampling approach of the ASV matrices with the *rrarefy* function of vegan [49, 50]. Differences in beta-diversity were assessed by principal component analysis (PCoA) [51] and canonical analysis principal coordinates (CAP) constrained by the significant factors [52], respectively, using the *cmdscale* function in vegan and *CAPdiscrim* function in BiodiversityR v.2.14.2.1 [53]. The effects of soil type, waterlogging stress, and soybean genotype on beta-diversity were quantified by multivariate permutational analysis of variance (PERMANOVA) with the *adonis2* function in vegan and 9999 permutations. Pairwise tests between factor levels were performed using the R package pairwiseAdonis v.0.4 [54]. Homogeneity of variance was checked using permutational analysis of multivariate dispersion (PERMDISP) [55] implemented as the *betadisper* function in vegan. The data were visualized in base R.

Genotype-dependent effects of waterlogging on individual ASVs and higher-level taxonomic groups were assessed using univariate PERMANOVA on the means of the 100-fold subsampled ASV matrices [39, 56]. Adjustment for multiple testing was performed using *q*-values [57] with the R package qvalue v.2.16.0 [58]. The taxonomic trees displaying the responsive ASVs were generated with iTol v6.1.2 [59] based on a tree matrix retrieved form the taxonomy table using the *taxa2dist* function from the vegan package and the *hclust* function from the ade4 v.1.7.20 package [60], respectively.

## Results

### Waterlogging effects on plant performance and soil chemistry

**Fig. 1:**
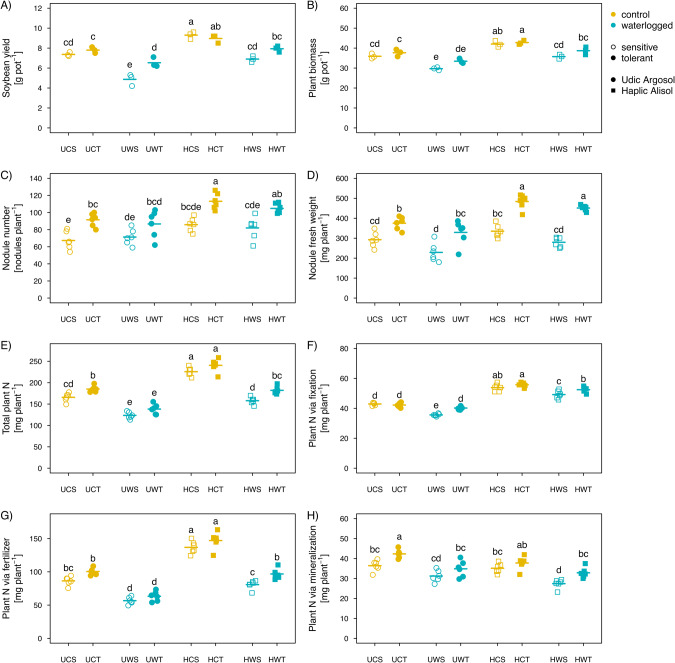
Effects of waterlogging on plant properties. Changes in soybean dry weight seed yield (**A**) and dry weight biomass (**B**) at harvest, number of root nodules (**C**) and nodule fresh weight (**D**), and total shoot N content (**E**) and its fractions derived from symbiotic N-fixation (**F**), N-fertilizer (**G**), and soil N mineralization (**H**) across both genotypes (tolerant vs. sensitive) and soils (Udic Argosol vs. Haplic Alisol). Different letters indicate significant (*p* < 0.05, *n* = 3 for yield and biomass, *n* = 6 for all others) differences as determined by Tukey’s HSD. U Udic Argosol, H Haplic Alisol, C control, W waterlogging, S sensitive genotype, T tolerant genotype.

Waterlogging had a significant (*p* < 0.0001) and genotype-dependent effect on plant dry biomass and seed yield at harvest stage (Fig. 1 and Supplementary Table S2). Waterlogging decreased seed yield of both the tolerant (QH34) and sensitive genotype (JD17) by 13.4% (±1.3 SE) and 24.5% (±3.7 SE) in the Haplic Alisol and 20.4% (±3.3 SE) and 32.3% (±4.7 SE) in the Udic Argosol, respectively (Fig. 1A). When both genotypes combined, seed yield decreased significantly due to waterlogging by an average of 18.9% (±3.3 SE) and 26.3% (±4.5 SE) in the Udic Argosol and Haplic Alisol, respectively (*p* < 0.05, Fig. 1A). Plant dry biomass decreased (*p* < 0.0001) due to waterlogging by an average of 14.1% (±3.2 SE) and 12.2% (±2.5 SE) in the Udic Argosol and Haplic Alisol, respectively (Fig. 1B).

Waterlogging did not affect the number of root nodules, but overall reduced nodule fresh weight in both soils (Supplementary Table S2). The tolerant genotype consistently carried more nodules (*p* < 0.0001) and had higher nodule weights (*p* < 0.0001), also without the waterlogging stress (Fig. 1C, D). These changes were not genotype dependent (Supplementary Table S2). Moreover, total plant N content and the N fractions derived from symbiotic fixation, fertilizer application, and soil mineralization of both soybean genotypes decreased in both soils due to waterlogging (Fig. 1E–H and Supplementary Table S2), but values were higher in the Haplic Alisol, except for N derived from soil mineralization (Fig. 1H). Waterlogging had a significant genotype-dependent effect on plant N derived from symbiotic fixation (Fig. 1F and Supplementary Table S2). Under waterlogging stress, the tolerant genotype had higher N contents in these different fractions except the fertilizer-derived and mineralized N in the Udic Argosol (Fig. 1F–H).

**Fig. 2:**
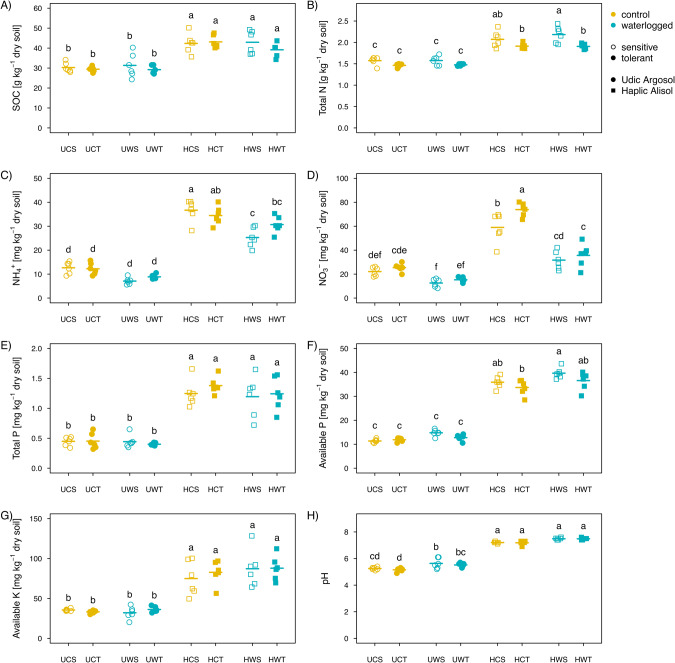
Effects of waterlogging on soil chemical properties. Changes in soil organic carbon (**A**), total nitrogen (**B**), ammonium (**C**), nitrate (**D**), total phosphorus (**E**), available phosphorus (**F**), available potassium (**G**), and pH (**H**) across both genotypes (tolerant vs. sensitive) and soils (Udic Argosol vs. Haplic Alisol). Different letters indicate significant (*p* < 0.05, *n* = 6) differences as determined by Tukey’s HSD. A acidic soil, N neutral soil, C control, W waterlogging, S sensitive genotype, T tolerant genotype.

Several soil properties changed under waterlogging stress (Fig. 2 and Supplementary Table S3). In general, waterlogging decreased NH_4_^+^ concentration in the rhizosphere soil of the sensitive genotype by 30.7% (±3.9 SE), and decreased NO_3_^−^ concentration in the rhizosphere soil of both the tolerant and sensitive genotypes by 50.9% (±6.6 SE) and 45.7% (±3.8 SE), in the Haplic Alisol (Fig. 2). Moreover, waterlogging increased soil pH of both the tolerant and sensitive genotype by 6.6% (±1.7 SE) and 6.4% (±3.0 SE) in the Udic Argosol. Only the changes in NH_4_^+^ concentration in the rhizosphere were genotype dependent. Other soil chemical parameters such as soil organic carbon, total nitrogen, total phosphorus, and available potassium, were not affected by waterlogging (Fig. 2 and Supplementary Table S3).

### Waterlogging effects on the rhizosphere microbiome

**Fig. 3:**
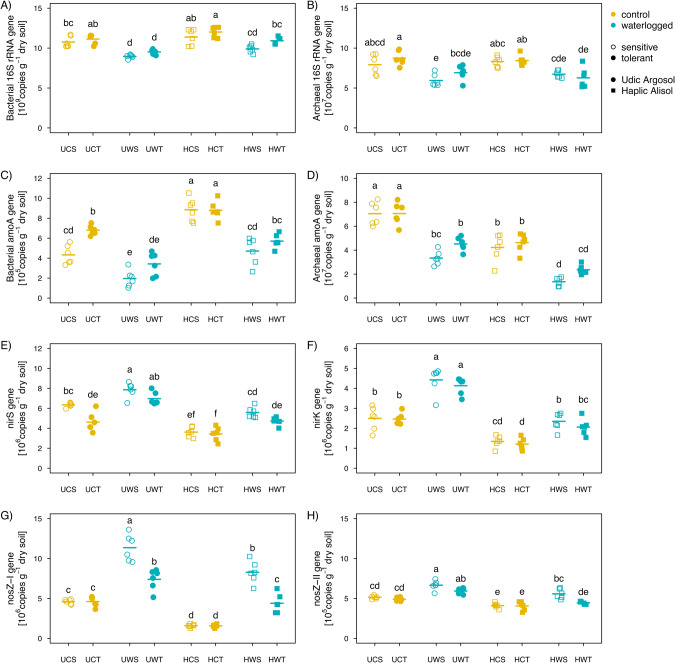
Effects of waterlogging on prokaryotic gene copy numbers. Changes in copy numbers of bacterial (**A**) and archaeal (**B**) 16S rRNA genes, bacterial (**C**) and archaeal (**D**) *amoA* genes, *nirS* (**E**) and *nirK* (**F**) genes, and *nosZ* clade I (**G**) and clade II (**H**) genes across both genotypes (tolerant vs. sensitive) and soils (Udic Argosol vs. Haplic Alisol). Different letters indicate significant (*p* < 0.05, *n* = 6) differences as determined by Tukey’s HSD. U Udic Argosol, H Haplic Alisol, C control, W waterlogging, S sensitive genotype, T tolerant genotype.

Soil type, waterlogging and genotype significantly influenced the copy numbers of ribosomal and N-cycling genes (Supplementary Table S4). Waterlogging reduced the copy numbers of the bacterial and archaeal 16S rRNA and *amoA* genes (AOB and AOA) and increased the copy numbers of the *nirS* and *nirK* as well as *nosZ* I and II genes in the rhizosphere soil (Fig. 3). The archaeal *amoA* and *nosZ* genes revealed genotype-dependent effects of waterlogging (Supplementary Table S4), showing generally higher *amoA* and lower *nosZ* genes copies in the rhizosphere soil under the tolerant compared to the sensitive genotype (Fig. 3). The bacterial *amoA* gene copy numbers were higher in the Haplic Alisol, whereas the archaeal *amoA*, *nirS*, *nirK* and *nosZ* gene copy numbers showed the opposite trend (Fig. 3).

**Fig. 4:**
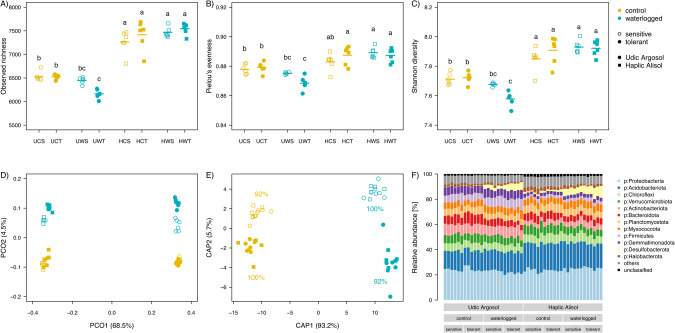
Effects of waterlogging on rhizosphere prokaryotic diversity. Changes in prokaryotic alpha- and beta-diversity in the soybean rhizosphere, i.e., observed richness (**A**), Pielou’s evenness (**B**), Shannon diversity (**C**), principal coordinate analysis (PCO) based on Bray–Curtis dissimilarities (**D**), canonical analysis of principal coordinates (CAP) constrained by treatment and genotype (**E**), and relative abundances of the major phyla (**F**). All metrics are based on iteratively rarefied ASV counts. Different letters in (**A**–**C**) indicate significant (*p* < 0.05, *n* = 6) differences as determined by Tukey’s HSD. Percent explained variance of each PCO axis (**D**) and percent between group variation of each CAP axis (**E**) are provided in parentheses. The CAP reclassification success rates (i.e., a quantitative estimation of the robustness of each treatment × genotype group) is provided next to the data clouds (**E**). The 12 phyla with the highest relative abundance are displayed, whereas less abundant phyla are grouped into “others”. ASVs not assigned at the phylum level (unclassified bacteria and archaea) are grouped into “unclassified”. U Udic Argosol, H Haplic Alisol, C control, W waterlogging, S sensitive genotype, T tolerant genotype.

Metabarcoding yielded a total of 1,886,813 (range 37,183–40,741) high-quality 16S rRNA gene sequences clustered into 16,442 ASVs. The rarefaction curves indicated sufficient coverage for subsequent assessment of alpha and beta diversity (Supplementary Fig. S2). Waterlogging had a significant genotype-dependent effect on alpha diversity (Table 1). Waterlogging tended to reduce alpha diversity in the rhizosphere soil of the tolerant genotype when compared to the sensitive genotype in the Udic Argosol but not in the Haplic Alisol (Fig. 4A–C). Alpha diversity did not differ between the genotypes in the unstressed control. Waterlogging also altered beta diversity in the rhizosphere (Table 1 and Fig. 4D, E). The major differences in prokaryotic community structure were attributed to soil type (68% of the explained variance), with a smaller amount explained by waterlogging (4%) and genotype (1%). Variation in beta diversity constrained by waterlogging and genotype to remove the overriding soil type effect revealed these underlying effects (Fig. 4E), which also showed a small but significant genotype-dependence of the waterlogging effect (Table 1). When testing each soil separately, both soils showed significant (*p* < 0.05) effects of waterlogging stress and soybean genotype on beta-diversity; however, only the Udic Argosol showed a genotype-dependent response (= significant interaction term) to waterlogging.

**Table 1:** Effects of soil type, waterlogging, genotype and their interactions on soil microbial alpha and beta diversity assessed by factorial ANOVA (alpha diversity) and PERMANOVA (beta diversity).

Factor	ɑ-diversity			β-diversity
	Richness	Evenness	Shannon	Bray–Curtis
	*F*(*P*)	*F*(*P*)	*F*(*P*)	*F*(*P*)
Soil (S)	**477.0 (<0.0001)**	**84.9 (<0.0001)**	**209.1 (<0.0001)**	**123.4 (0.0001)**
Treatment (T)	0.3 (0.5688)	2.2 (0.1491)	1.8 (0.1915)	**7.6 (0.0001)**
Genotype (G)	0.1 (0.7851)	0.3 (0.5703)	0.3 (0.5741)	**2.1 (0.0023)**
S × T	**17.5 (0.0002)**	**15.3 (0.0003)**	**18.4 (0.0001)**	**2.6 (0.0005)**
S × G	**7.8 (0.0080)**	2.6 (0.1133)	**4.7 (0.0364)**	**1.7 (0.0133)**
T × G	3.7 (0.0622)	**8.2 (0.0065)**	**7.5 (0.0092)**	**1.5 (0.0341)**
S × T × G	1.2 (0.2715)	0.1 (0.7815)	0.4 (0.5229)	**1.4 (0.0398)**

Values represent *F*-ratio (*F*) and level of significance (*P*).

Bold font indicates statistical significance.

Proteobacteria (23.8 ± 0.9% relative abundance), Acidobacteriota (18.3 ± 2.2%), Chloroflexi (6.4 ± 0.6%), Verrucomicrobiota (6.4 ± 0.7%), Actinobacteriota (6.2 ± 2.1%), Bacteroidota (5.7 ± 0.8%), Planctomycetota (5.6 ± 0.5%), and Myxococcota (5.1 ± 0.3%) were the predominant bacterial phyla with ≥5% relative abundance (Fig. 4F). A total of 25 phyla responded significantly (*q* < 0.1) to waterlogging in one or both soils, with 10 phyla responding consistently in both soils by either decreasing (Bacteroidota, Bdellovibrionota, Dependentiae, Elusimicrobiota, FCPU426, Nitrospirota, and Planctomycetota) or increasing (Desulfobacterota, Myxococcota, and WS4) in waterlogged soils. Acidobacteriota and Proteobacteria were also significantly altered by waterlogging but showed contrasting responses in the two soils by increasing in the waterlogged Udic Argosol but decreasing in the waterlogged Haplic Alisol.

**Fig. 5:**
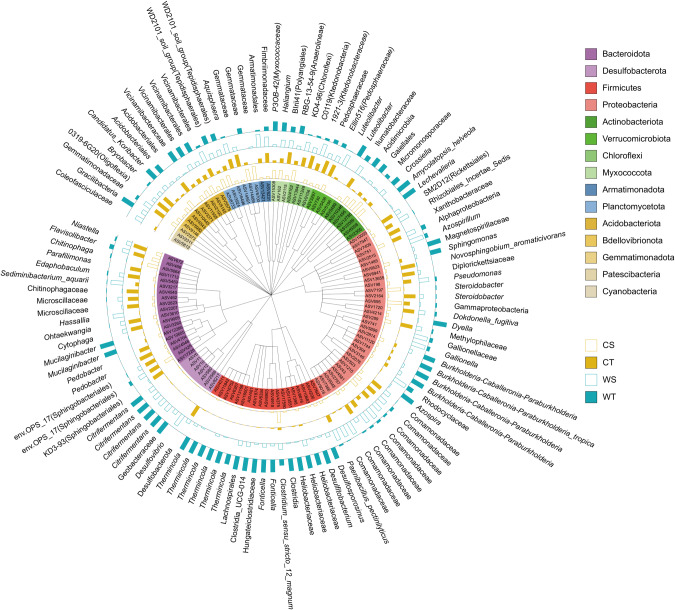
Prokaryotic taxa showing a genotype-dependent response to waterlogging. Taxonomic tree showing the prokaryotic ASVs assigned at the last common assignment level that responded significantly to interaction between genotype and waterlogging in the Udic Argosol (PERMANOVA, *q* < 0.1, *n* = 6). Bars represent scaled relative abundances of ASVs that are proportionally enriched under the control and waterlogging treatments in the rhizosphere of the sensitive and tolerant genotypes in the Udic Argosol. The outer circle shows the last common assignment level information of the ASVs. C control, W waterlogging, S sensitive genotype, T tolerant genotype.

In the following, we explicitly focus on taxa showing a significant (*q* < 0.1) genotype-dependent effect of waterlogging, and only for the Udic Argosol that showed a significant (*p* < 0.05) interaction term between waterlogging and genotype in the beta-diversity analysis. However, the response of all detected taxa from phylum to ASV level is available as Supplementary Data 1. A total of 115 ASVs representing 3.2% of the sequences showed a genotype-dependent response to waterlogging and these ASVs were broadly spread across the taxonomic tree (Fig. 5). ASVs that were enriched under waterlogging and primarily associated with the tolerant genotype were assigned to the taxa *Mucilaginibacter* (Bacteroidota), *Citrifermentans* (Desulfobacterota), *Thermincola, Fonticella, Desulfosporosinus*, and Heliobacteriaceae (Firmicutes), *Azospira*, *Burkholderia-Caballeronia-Paraburkholderia, Dyella*, Magnetospirillaceae, and *Sphingomonas* (Proteobacteria), *Lechevalieria, Amycolatopsis* (Actinobacteriota), *Luteolibacter* (Verrucomicrobiota), *Haliangium* (Myxococcota), Vicinamibacterales (Acidobacteriota), *Candidatus Koribacter* (Actinobacteriota) and Gracilibacteria (Patescibacteria).

## Discussion

This study revealed how different soybean genotypes—that were shown to be either tolerant or sensitive to waterlogging stress—modulate N acquisition and their associated rhizosphere microbiome after 3 days of experimental waterlogging in two types of soil. Results showed that plant N uptake, seed yield and enriched microbial taxa show genotype-dependent responses to waterlogging that differ between the Udic Argosol and Haplic Alisol soil. This confirmed our hypotheses that the tolerant genotype can maintain higher levels of N uptake and associates with specific prokaryotic communities in the rhizosphere that are related to N-cycling.

### Effects of waterlogging on plant N origins and yield

Waterlogging stress reduced the soybean uptake of N derived from symbiotic N_2_ fixation and fertilizer (Fig. 1F, G), which are both critical sources for yield gain. The reduced total N uptake ultimately reduced soybean yield in both soil types (Fig. 1). This might partially be attributed to two reasons. Firstly, a sharp decline in oxygen availability in waterlogged soils can reduce root respiration and nodulation with direct consequences for N uptake. Secondly, waterlogging increases denitrification rates, which can lead to the emission of nitrogen from soil in the form of N_2_O and N_2_, resulting in lower nitrogen availability for the plants. In this study, 3 days of waterlogging reduced the abundance of prokaryotes involved in aerobic processes like nitrification and promoted prokaryotes involved in anaerobic processes like denitrification (Fig. 3), which might have ultimately triggered N losses from the soil (Fig. 2) and reduced plant N uptake (Fig. 1). This is in agreement with previous studies looking at waterlogging effects on microbial processes [61–63]. The reduction of N absorption by plants was more pronounced in the Udic Argosol than in the Haplic Alisol (Fig. 2). Compared to the Haplic Alisol, the Udic Argosol had lower porosity and higher clay content, thereby restricting water and air movement in the soil and generating a greater lack of oxygen and an accumulation of carbon dioxide, which might aggravate the effects of waterlogging [64–66]. Overall, these results demonstrated that waterlogging had a more pronounced negative impact on seed yield in the Udic Argosol rather than in the Haplic Alisol (Fig. 1B).

The extent of reduced N uptake caused by the waterlogging differed between the soybean genotypes, with the sensitive genotype exhibiting lower N uptake than the tolerant one (Fig. 1E). Higher nodule fresh weight of the tolerant genotype could be one reason for this effect (Fig. 1D). A higher load of rhizobia can lead to higher symbiotic N fixation, which in turn might sustain N supply even under waterlogging [67]. Indeed, N derived through symbiotic fixation under waterlogging was higher in the tolerant compared to the sensitive genotype (Fig. 1F).

In addition, the primary and secondary metabolism in soybean roots could be strongly affected by waterlogging stress [8]. A previous study showed that much of the altered metabolism was related to carbon and nitrogen turnover in general, and the phenylpropane pathway that is important for soybean quality in particular, and these metabolic patterns were different between waterlogging-tolerant and -sensitive soybean genotypes [8]. It is worth noting that although waterlogging did not affect plant biomass differently between the two genotypes, there was a significant difference in yield at maturity, suggesting that the waterlogging during flowering likely had a delayed effect on N uptake. This phenomenon can be partly attributed to that plants subjected to stress in pots often exhibit less severe recovery ability than those in the field [68]. In addition, in this study, waterlogging was performed on soybean during its critical flowering period for nodule formation. Short-term waterlogging at this stage can adversely affect soybean pollination, flower abscission, rhizobial infection, nodule formation, and even lead to nodule death in severe cases, which could reduce biological nitrogen fixation and yield [69]. This is similar to the study of Wang et al. [70] who observed that 2 days of waterlogging caused stagnation of morphological development (plant heights and leaf areas) and reduced cotton yield in the long-term. In addition, a previous study found that the soybean genotype tolerant to waterlogging were able to restore their metabolite concentrations to pre-waterlogging levels and recover their enzyme activities faster than the sensitive genotype [71].

### Effects of waterlogging on ammonia-oxidizing and denitrifying guilds

The reduced plant N immobilization upon waterlogging might also be due to increased losses of inorganic N from the system through rhizosphere microorganisms. In this study, irrespective of soil types and soybean genotypes, waterlogging reduced the abundance of ammonia oxidizers (bacterial and archaeal *amoA* genes), while an opposite trend was observed for the denitrifiers (*nirS*, *nirK* and *nosZ* I and II genes). This finding is consistent with previous studies which have reported a decrease of ammonia oxidizers under increased soil moisture [13, 72]. Soil nitrifying microorganisms depend on oxygen availability and are usually negatively affected by waterlogging [73]. In contrast, soil denitrifying microorganisms necessitate nitrate and usually get stimulated in waterlogged conditions [74]. Compared with the sensitive genotype, the rhizosphere soil from the tolerant genotype carried higher loads of ammonium oxidizers and lower loads of denitrifiers (Fig. 3). These results indicate that the tolerant genotype may obtain more plant-available nitrate, resulting in a better nutrition of the plant [13]. These observations are in line with the higher NH_4_^+^ and NO_3_^−^ levels in the rhizosphere soil of the tolerant genotype after waterlogging stress (Fig. 2).

### Effects of waterlogging on rhizosphere prokaryotic communities

Plants can associate with beneficial microorganisms to alleviate stress [75, 76]. In this study, waterlogging caused changes in the rhizosphere environment and affected the diversity and structure of rhizosphere microorganisms on both types of soil. Changes in microbial key players such as N cycling guilds may differentially affect N absorption of the two genotypes in the two soils.

ASVs assigned to known anaerobic genera such as *Citrifermentans* (formerly known as *Geobacter* and later as *Geomonas* [77, 78] henceforth referred to as *Geobacter*/*Geomonas* complex to allow better comparison with other studies) and *Desulfosporosinus* were enriched in the rhizosphere of the tolerant genotype under waterlogging conditions in Udic Argosol (Fig. 5). These results are in line with previous studies showing an increase of these taxa in oxygen-limited soils such as compacted arable [39] and forest [79] soils, paddy rice soils [80, 81] and water-logged agricultural fields [82]. Studies have shown that the loss of N-fixing ability of plants is related to a proportional reduction of potentially diazotrophic *Geobacter* species [83, 84]. Another study on soybeans showed that *Geobacter* was positively correlated with N_2_ fixation [85]. *Geobacter* species might be predominant N-fixers in paddy rice fields [86]. Moreover, *Geobacter/Geomonas*, and *Desulfosporosinus* species occupy an important ecological niche in anaerobic environments, and considered to be responsible for microbial Fe(III) reduction [87, 88], which can also influence the cycling of other compounds such as N or phosphorus (P) [89, 90]. For example, nitrate-dependent anaerobic Fe redox cycling of ammonium could produce ammonia, which can be used as a nutrient for plant production [90]. Under dry conditions, Fe(II) is oxidized to produce FeOOH (a form of Fe[III]), which can bind P. However, under anoxic conditions, Fe[III] is reduced to Fe(II) by anaerobic respiration, releasing FeOOH-bound P into the soil [89].

Other taxa that were enriched under waterlogging in association with the tolerant genotype included *Candidatus Koribacter*, *Lechevalieria*, *Mucilaginibacter*, *Burkholderia-Caballeronia-Paraburkholderia*, and *Azospira* (Fig. 5). Conversely, the sensitive genotype showed an enrichment of the Comamonadaceae family under waterlogging conditions in the Udic Argosol (Fig. 5). Some of the microorganisms affiliated to these genera have been related to N cycling. For example, *Burkholderia-Caballeronia-Paraburkholderia* [91], *Azospira* [92] and *Sphingomonas* [93] are known N-fixing bacteria. *Mucilaginibacter* [94]*, Haliangium* [95] and *Lechevalieria* [96] have been reported as plant growth promoting bacteria that produce large amounts of extracellular polysaccharides and beneficial to the growth of plants. Similar to *Geobacter*, *Candidatus Koribacter* may be a fermentative iron-reducing bacterium [97, 98]. Additionally, Comamonadaceae family has been previously reported in microbial consortia used for denitrification [99]. Notably, although the Rhizobiales were negatively affected by waterlogging, none of taxa related to rhizobia such as *Rhizobium*, *Bradyrhizobium* and *Mesorhizobium* showed a differential response between the tolerant and sensitive genotypes (Supplementary Data 1). Overall, the strong enrichment of the above-mentioned taxa under waterlogged conditions and primarily associated with the tolerant or sensitive genotype might be an indication of increased N-fixation and less N_2_O emissions, and thus better N and other nutritional supply for the plants.

## Conclusions

A short waterlogging period of 3 days altered soybean N absorption and rhizosphere microbiome structure, reducing yield in the range of 13 to 32%. This could partially be attributed to a reduced acquisition of nitrogen derived from symbiotic fixation, fertilizer, and soil mineralization. The tolerant soybean genotype Qihuang34 revealed reduced stress symptoms compared to the sensitive genotype Jidou17, showing increased nodulation and N uptake, and an altered rhizosphere microbiome structure including the absolute and relative abundance of N-cycling guilds. The tolerant genotype harbored more ammonia oxidizers and less nitrous oxide reducers compared to the sensitive genotype under waterlogging. Furthermore, anaerobic, nitrogen-fixing, denitrifying and iron-reducing bacteria such as *Geobacter/Geomonas*, *Desulfosporosinus*, *Sphingomonas* and *Candidatus Koribacter* were proportionally enriched in association with the tolerant genotype under waterlogging. These changes of the rhizosphere microbiome might ultimately help the plant to improve N uptake under waterlogged and largely anoxic conditions. These effects were soil type dependent, calling for a broader investigation to obtain more universally valid conclusions. Ultimately, this study provides new evidence for the response of ammonia oxidizers and denitrifiers in the rhizosphere of different soybean genotypes to waterlogging, and provides a theoretical basis for the importance of root-associated microbial communities in improving N use efficiency under waterlogging.

## Supplementary information

Supplementary Information

Supplementary Data 1

## Data Availability

Raw sequence data are available at the NCBI sequence read archive (SRA) under the accession number PRJNA723464.
