# Supplementary material for: Potential relevance between soybean nitrogen uptake and rhizosphere prokaryotic communities under waterlogging stress
Source: ISME Commun. 2023 Jul 11;3:71. doi: 10.1038/s43705-023-00282-0 (PMC10336055; doi:10.1038/s43705-023-00282-0)
Supplement: Supplementary file 1 — Supplementary Information [file 43705_2023_282_MOESM1_ESM.pdf]

Potential relevance between soybean nitrogen uptake and rhizosphere prokaryotic communities under waterlogging stress

Tengxiang Lian[1,2,3]*, Lang Cheng[1,2], Qi Liu[1,2], Taobing Yu[1,2], Zhandong Cai[1,2], Hai Nian[1,2]* and Martin Hartmann[3]*

[1] The State Key Laboratory for Conservation and Utilization of Subtropical Agro-bioresources, South China Agricultural University, Guangzhou, Guangdong, China.

[2] The Key Laboratory of Plant Molecular Breeding of Guangdong Province, College of Agriculture, South China Agricultural University, Guangzhou, Guangdong, China.

[3] Institute of Agricultural Sciences, ETH Zurich, Zurich, Switzerland

***Corresponding authors**:

Dr. Tengxiang Lian

Address: No.483 Wushan Road, Guangzhou, Guangdong, 5642, China,

Tel: +86 02085288024, Fax: +86 02085288024, E-mail: liantx@scau.edu.cn

Dr. Hai Nian

Address: No.483 Wushan Road, Guangzhou, Guangdong, 510642, China,

Tel: +86 02085288024, Fax: +86 02085288024, E-mail: hnian@scau.edu.cn

Dr. Martin Hartmann

Address: LFH B6, Universitätsstrasse 2, 8092 Zürich, Switzerland,

Tel: +41-44-633-8836, E-mail: martin.hartmann@usys.ethz.ch

Table S1. Primer sequence and PCR cycling protocols.

Target	Primer name	Primer sequence 5’-3’	Cycling program	Reference
Plasmid	SP6<br>T7	TACGATTTAGGTGACACTATAG<br>TAATACGACTCACTATAGGG	1 x (95 °C for 60 s)<br>30 x (94 °C for 1 min, 55 °C for 1 min, 75 °C for 2 mins)<br>1 x (75 °C for 5 min)	[1]
Bacterial 16S rRNA	515F<br>909R	Barcode+(GTGCCAGCMGCCGCGG)<br>Barcode+(CCGTCAATTCMTTTRAGTTT)	1 x (95 °C for 60 s)<br>30 x (94 °C for 1 min, 55 °C for 1 min, 75 °C for 2 mins)<br>1 x (75 °C for 5 min)	[2]
Archaeal 16S rRNA	967F<br>1060R	GGCCATGCACCWCCTCTC<br>ACGATGCGACGCCGCGTG	1 x (95 °C for 60 s)<br>30 x (94 °C for 1 min, 55 °C for 1 min, 75 °C for 2 mins)<br>1 x (75 °C for 5 min)	[3]
AOB amoA	amoA-1F<br>amoA-2R	GGGGTTTCTACTGGTGGT<br>CCCCTCKGSAAAGCCTTCTTC	1 x (95 °C for 10 mins)<br>39 x (94 °C for 45 s, 58 °C for 45 s, 72 °C for 45 s)<br>1 x (95 °C for 15 s)	[4]
AOA amoA	CrenamoA23F<br>CrenamoA616R	ATGGTCTGGCTWAGACG<br>GCCATCCATATGTATGTCCA	1 x (95 °C for 10 mins)<br>39 x (94 °C for 45 s, 55 °C for 45 s, 72 °C for 45 s)<br>1 x (95 °C for 15 s)	[4]
nirS	nirS-efF<br>nirS-efR	CACCCGGAGTTCATCGTC<br>ACCTTGTTGGACTGGTGGG	1 x (95 °C for 10 mins)<br>39 x (94 °C for 45 s, 60 °C for 45 s, 72 °C for 45 s)<br>1 x (95 °C for 15 s)	[5]
nirK	nirKC2F<br>nirKC2R	TGCACATCGCCAACGGNATGTWYGG<br>GGCGCGGAAGATGSHRTGRTCNAC	1 x (95 °C for 10 mins)<br>39 x (94 °C for 45 s, 60 °C for 45 s, 72 °C for 45 s)<br>1 x (95 °C for 15 s)	[6]
*nosZ*I	NosZ2F<br>NosZ2R	CGCRACGGCAASAAGGTSMSSGT<br>CAKGTRCAKSGCRTGGCAGAA	1 x (95 °C for 15 mins)<br>6 x touch down -1 °C/cycle (95 °C for 15 s, 65 °C for 30 s)<br>40 x (95 °C for 15 s, 60°C for 15 s, 72°C for 30 s, 80°C for 15 s)<br>1 x (95 °C for 30 s)	[7]
*nosZ*II	nosZIIF<br>nosZIIR	TIGGICCIYTKCAYAC<br>GCIGARCARAAITCBGTRC	1 x (95 °C for 15 mins)<br>6 x touch down -1 °C/cycle (95 °C for 15 s, 65 °C for 30 s)<br>40 x (95 °C for 15 s, 60°C for 15 s, 72°C for 30 s, 80°C for 15 s)<br>1 x (95 °C for 30 s)	[8]

Table S2. Effects of soil type, waterlogging stress, and soybean genotypes on plant yield and biomass, the number of nodules and nodule fresh weight, and the total shoot N content including its fractions derived from N fixation, fertilization and mineralization as assessed by factorial ANOVA. Values indicate the F-ratio (F) and the level of significance (*P*).

	Yield	Biomass	Nodules	Nodule weight	N (total)	N (fixation)	N (fertilization)	N (mineralization)
	F(P)	F(P)	F(P)	F(P)	F(P)	F(P)	F(P)	F(P)
Soil (S)	**107.0 (<0.0001)**	**100.8 (<0.0001)**	**33.2 (<0.0001)**	**55.4 (<0.0001)**	**278.2 (<0.0001)**	**657.3 (<0.0001)**	**286.6 (<0.0001)**	**12.6 (0.0010)**
Treatment (T)	**130.0 (<0.0001)**	**85.9 (<0.0001)**	1.2 (0.2842)	**20.7 (<0.0001)**	**344.0 (<0.0001)**	**77.8 (<0.0001)**	**360.6 (<0.0001)**	**59.2 (<0.0001)**
Genotype (G)	19.7 **(0.0004)**	**16.8 (0.0008)**	**56.1 (<0.0001)**	**133.7 (<0.0001)**	**39.6 (<0.0001)**	**21.7 (<0.0001)**	**26.4 (<0.0001)**	**28.7 (<0.0001)**
SxT	0.3 (0.6049)	0.0 (0.9767)	0.9 (0.3496)	0.2 (0.6234)	**10.3 (0.0026)**	0.5 (0.4840)	**18.5 (0.0001)**	0.0 (0.9958)
SxG	**4.9 (0.0415)**	0.6 (0.4344)	0.8 (0.3784)	**10.0 (0.0030)**	0.2 (0.6685)	0.5 (0.5009)	0.4 (0.5374)	0.2 (0.6598)
TxG	**16.9 (0.0008)**	3.5 (0.0797)	1.2 (0.2722)	0.9 (0.3573)	0.2 (0.6747)	**11.3 (0.0017)**	0.0 (0.8550)	0.0 (0.8753)
SxTxG	0.0 (0.8355)	0.0 (0.9069)	0.1 (0.7194)	0.0 (0.9022)	1.4 (0.2420)	**4.2 (0.0479)**	2.1 (0.1546)	2.4 (0.1288)

Table S3. Effects of soil type, waterlogging stress, and soybean genotypes on soil chemical properties including soil organic carbon (SOC), total nitrogen (TN), ammonium (NH_4^+), nitrate (NO_3^-), total phosphorus (TP), available phosphorus (Olsen-P), available potassium (AK), and pH, as assessed by factorial. Values indicate the F-ratio (F) and the level of significance (*P*).

	SOC	TN	NH_4^+	NO_3^-	TP	Olsen-P	AK	pH
	F(P)	F(P)	F(P)	F(P)	F(P)	F(P)	F(P)	F(P)
Soil (S)	**107.9 (<0.0001)**	**236.3 (<0.0001)**	**568.5 (<0.0001)**	**257.8 (<0.0001)**	**227.4 (<0.0001)**	**1396.3 (<0.0001)**	**152.8 (<0.0001)**	**1525.3 (<0.0001)**
Treatment (T)	0.3 (0.5827)	0.9 (0.3526)	**44.4 (<0.0001)**	**120.2 (<0.0001)**	1.3 (0.2693)	**18.5 (0.0001)**	1.2 (0.2898)	**44.8 (<0.0001)**
Genotype (G)	1.7 (0.1973)	**25.2 (<0.0001)**	1.5 (0.2212)	**10.2 (0.0028)**	0.4 (0.5256)	**7.2 (0.0105)**	0.4 (0.5201)	1.4 (0.2432)
SxT	0.9 (0.3587)	0.5 (0.4829)	3.0 (0.0921)	**34.8 (<0.0001)**	0.4 (0.5542)	0.9 (0.3614)	1.3 (0.2625)	0.7 (0.4185)
SxG	0.0 (0.9954)	3.1 (0.0881)	0.3 (0.6141)	2.7 (0.1059)	1.0 (0.3244)	2.2 (0.1457)	0.2 (0.6760)	1.0 (0.3309)
TxG	1.6 (0.2108)	0.7 (0.4074)	**7.4 (0.0097)**	2.2 (0.1464)	0.3 (0.5592)	1.8 (0.1849)	0.0 (0.9642)	0.0 (0.9868)
SxTxG	0.5 (0.4856)	1.0 (0.3296)	2.2 (0.1428)	1.8 (0.1906)	0.0 (0.8650)	0.4 (0.5163)	0.7 (0.3998)	0.0 (0.8814)

Table S4. Effects of soil type, waterlogging stress, and soybean genotype on gene copy numbers of bacterial and archaeal 16S rRNA and nitrogen cycling genes as assessed factorial ANOVA. Values represent F-ratio (F) and level of significance (P).

Factor	Bacterial 16S rRNA	Archaeal 16S rRNA	Bacterial amoA	Archaeal amoA	nirS	nirK	nosZ-I	nosZ-II
	F(P)	F(P)	F(P)	F(P)	F(P)	F(P)	F(P)	F(P)
Soil (S)	**32.1 (<0.0001)**	0.1 (0.8210)	**106.2 (<0.0001)**	**117.0 (<0.0001)**	**141.0 (<0.0001)**	**166.7 (<0.0001)**	**107.2 (<0.0001)**	**87.6 (<0.0001)**
Treatment (T)	**77.7 (<0.0001)**	**53.6 (<0.0001)**	**134.2 (<0.0001)**	**171.8 (<0.0001)**	**100.1 (<0.0001)**	**115.6 (<0.0001)**	**264.6 (<0.0001)**	**87.2 (<0.0001)**
Genotype (G)	**14.3 (0.0005)**	2.0 (0.1607)	**19.0 (<0.0001)**	**9.0 (0.0047)**	**26.4 (<0.0001)**	2.1 (0.1513)	**45.1 (<0.0001)**	**20.5 (<0.0001)**
SxT	1.4 (0.2371)	0.0 (0.9603)	1.8 (0.1924)	1.7 (0.2042)	0.8 (0.3913)	**11.7 (0.0014)**	0.0 (0.9899)	2.1 (0.1508)
SxG	1.1 (0.2985)	**4.3 (0.0454)**	**7.2 (0.0108)**	0.1 (0.8130)	**5.0 (0.0318)**	0.0 (0.8552)	0.0 (0.9696)	0.2 (0.6902)
TxG	0.7 (0.3990)	0.2 (0.6851)	0.0 (0.9741)	**4.2 (0.0476)**	0.1 (0.8088)	0.7 (0.4245)	**44.8 (<0.0001)**	**11.0 (0.0020)**
SxTxG	0.1 (0.7555)	0.5 (0.4766)	3.4 (0.0731)	0.4 (0.5126)	**4.5 (0.0409)**	0.0 (0.8450)	0.0 (0.9291)	1.6 (0.2122)

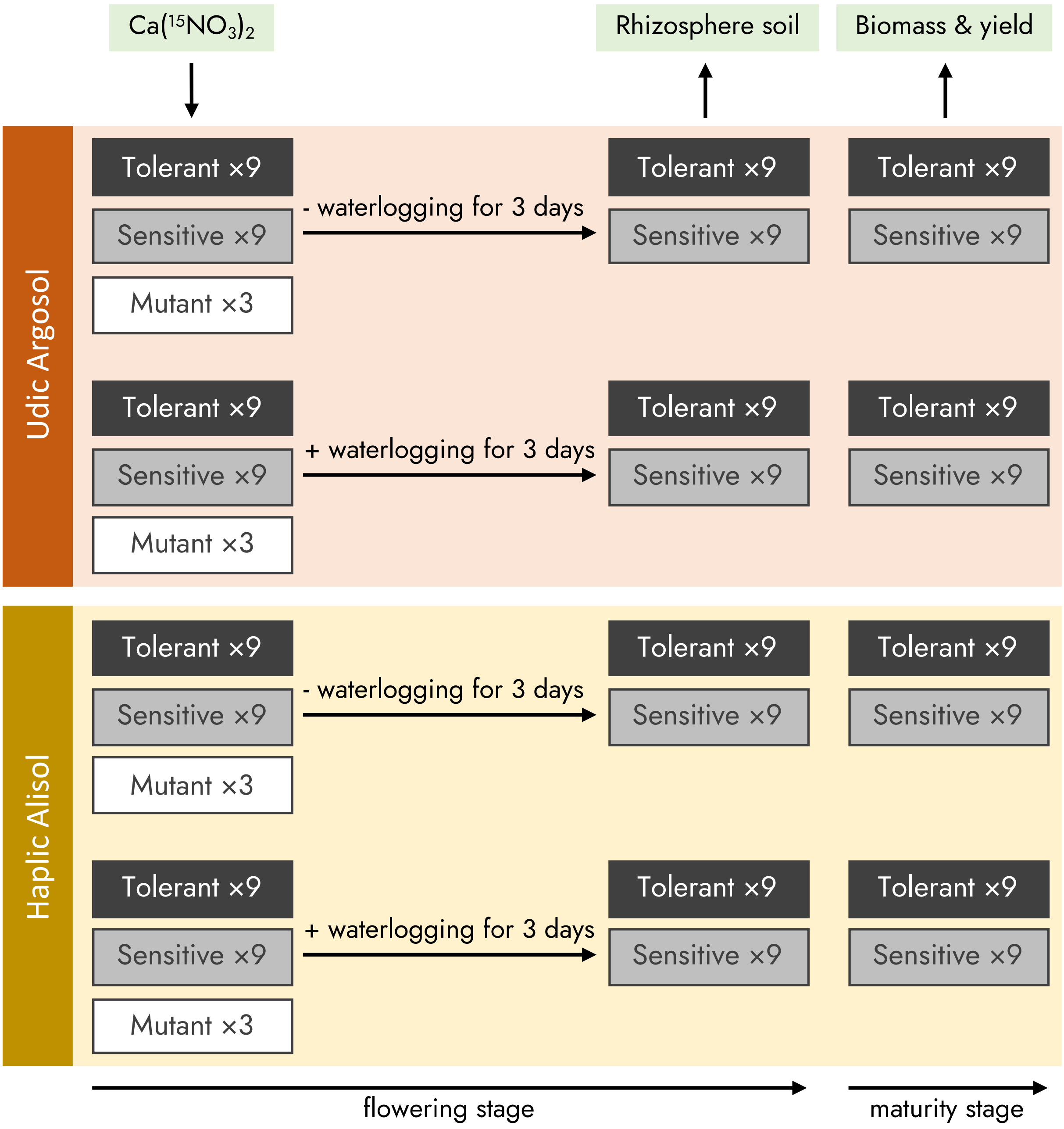

Supplementary Figure S1. The experimental design of investigating the response of two soybean genotypes to waterlogging stress in Udic Argosol and Haplic Alisol. The QH34 (waterlogging tolerant) and JD17 (waterlogging sensitive) soybean varieties were subjected to waterlogging of three days and compared to a non-waterlogged control. A soybean mutant incapable of inducing nodulation was planted under the different waterlogging conditions and soil types as reference species without the ability of fixing atmospheric N_2. Ca($^{15}NO_3$)$_2$ was added to the soil to assess N adsorption from different sources including N-fixation, N- fertilization and soil N-mineralization. Rhizosphere soil for microbial assessment was collected at flowering stage and soybean biomass and yield were measured at maturity stage.

Supplementary Figure S2. Rarefaction curves showing sequencing depth and ASV richness for all 48 samples.

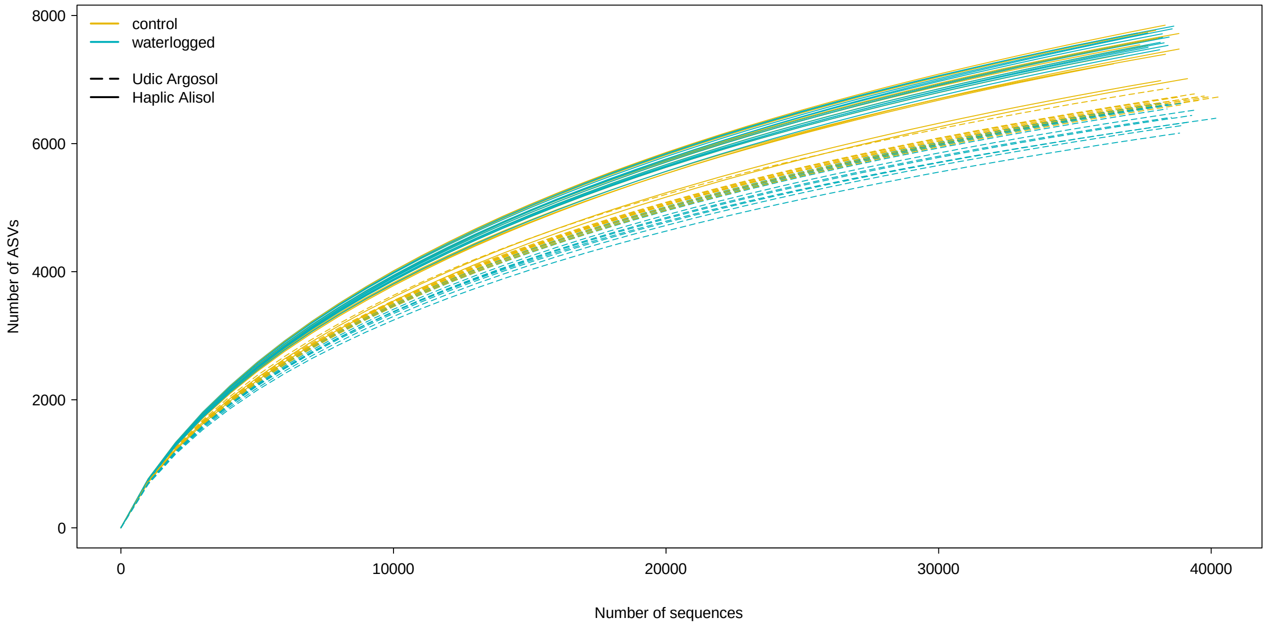

References

1. Longepierre M, Widmer F, Keller T, Weisskopf P, Colombi T, Six J, et al. Limited resilience of the soil microbiome to mechanical compaction within four growing seasons of agricultural management. ISME Commun. 2021; **1**: 1–13.
2. Caporaso JG, Lauber CL, Walters WA, Berg-Lyons D, Huntley J, Fierer N, et al. Ultra-high-throughput microbial community analysis on the Illumina HiSeq and MiSeq platforms. ISME J. 2012; **6**: 1621–1624.
3. Wang Y, Qian P-Y. Conservative fragments in bacterial 16S rRNA genes and primer design for 16S ribosomal DNA amplicons in metagenomic studies. PloS one 2009; **4**: e7401.
4. Hallin S, Jones CM, Schloter M, Philippot L. Relationship between N-cycling communities and ecosystem functioning in a 50-year-old fertilization experiment. ISME J. 2009; **3**: 597–605.
5. Smith CJ, Nedwell DB, Dong LF, Osborn AM. Diversity and abundance of nitrate reductase genes (*narG* and *napA*), nitrite reductase genes (*nirS* and *nrfA*), and their transcripts in estuarine sediments. Appl Environ Microb. 2007; **73**: 3612–3622.
6. Wei W, Isobe K, Nishizawa T, Zhu L, Shiratori Y, Ohte N, et al. Higher diversity and abundance of denitrifying microorganisms in environments than considered previously. ISME J. 2015; **9**: 1954–1965.
7. Henry S, Bru D, Stres B, Hallet S, Philippot L. Quantitative Detection of the nosZ Gene, Encoding Nitrous Oxide Reductase, and Comparison of the Abundances of 16S rRNA, narG, nirK, and nosZ Genes in Soils. Appl Environ Microb. 2006; **72**: 5181–5189.
8. Jones CM, Graf DR, Bru D, Philippot L, Hallin S. The unaccounted yet abundant nitrous oxide-reducing microbial community: a potential nitrous oxide sink. *ISME J.* 2013; **7**: 417–426.
